# Establishment of a Protocol for Viability qPCR in Dental Hard Tissues

**DOI:** 10.3390/microorganisms12071400

**Published:** 2024-07-11

**Authors:** Torsten Sterzenbach, Vanessa Neumann, Evelyn Trips, Sabine Basche, Christian Hannig, Marie-Theres Kühne

**Affiliations:** 1 Policlinic of Operative Dentistry, Periodontology and Pediatric Dentistry, Medical Faculty Carl Gustav Carus, Dresden University of Technology, 01307 Dresden, Germany; torsten.sterzenbach@ukdd.de (T.S.); vanessa.neumann@mailbox.tu-dresden.de (V.N.); sabine.basche@ukdd.de (S.B.); christian.hannig@ukdd.de (C.H.); 2 Coordination Centre for Clinical Trials, Faculty of Medicine Carl Gustav Carus, Dresden University of Technology, Fetscherstraße 74, 01309 Dresden, Germany

**Keywords:** viability qPCR, propidium monoazide, dental hard tissues, endodontics, *Enterococcus faecalis*, bacterial quantification

## Abstract

The aim of the study was to establish a live/dead qPCR with propidium monoazide (PMA) that can quantitatively differentiate between viable/non-viable microorganisms in dental hard tissues. Human premolars (*n* = 88) were prepared with nickel–titanium instruments and incubated with *E. faecalis* (21 d). Subsequently, the bacteria in half of the teeth were devitalized by heat inactivation (100 °C, 2 h). The following parameters were tested: PMA concentrations at 0 µmol (control), 50 µmol, 100 µmol, and 200 µmol; PMA incubation times of 30 min and 60 min, and blue light treatment for 30 min and 60 min. The teeth were ground using a cryomill and the bacterial DNA was quantified using qPCR, ANOVA, and *p* = 0.05. The qPCR of the control group detected a similar number of avital 9.94 × 10^6^ and vital 1.61 × 10^7^ bacterial cells. The use of PMA inhibited the amplification of DNA from non-viable cells during qPCR. As a result, the best detection of avital bacteria was achieved with the following PMA parameters: (concentration, incubation time, blue light treatment) 200-30-30; 5.53 × 10^4^ (avital) and 1.21 × 10^0.7^ (vital). The live/dead qPCR method using PMA treatment is suitable for the differentiation and quantification of viable/non-viable microorganisms in dentin, as well as to evaluate the effectiveness of different preparation procedures and antimicrobial irrigants in other biological hard substances.

## 1. Introduction

Endodontic treatments are very common in routine dental practice and have increased worldwide in recent years due to the aging population and the progress made in conservative dentistry. During endodontic treatment, both mechanical preparation of the contaminated root and different disinfective agents and irrigation protocols are typically used for the removal of microbial infections within the root. However, endodontic treatments can frequently fail due to incomplete removal of viable bacteria and the recurrence of microbial infections. Different analytical methods were traditionally used for evaluating the efficiency of various treatment protocols. Residual microorganisms can be removed directly out of root canals with paper points for the determination of colony-forming units (CFUs), giving an insight into the viability of the detected microorganisms [1,2]. However, the microorganisms collected by paper points mostly involve the bacteria from the root canal walls and not from the strongly branched side canals or dentinal tubules [3]. In addition, different microscopical methods like fluorescence microscopy, transmission electron microscopy, and scanning electron microscopy or confocal laser scanning microscopy are commonly used [4,5,6]. With fluorescent microscopy and confocal laser scanning microscopy, the viability of microorganisms can be differentiated by various valuable live/dead staining methods [7,8]. Moreover, for these methods, the teeth need to be sectioned in slices and the microorganisms stained, or the visualization depth and field of view will be limited and will not display the complete amount of bacteria within the root canal system [8]. Scanning electron microscopy and transmission electron microscopy can visualize the distribution, form, and architecture of microorganisms and biofilms well, though they do not distinguish between viable and non-viable bacteria [9,10,11]. Furthermore, various sensing technologies using biosensors have been established and will most likely play an even bigger role in the near future [12,13,14,15]. 

We recently published a method to quantify microbial infections within root canals via cryogenic milling of teeth and molecular biological quantification of bacteria via quantitative PCR (qPCR) [16]. This method was successfully applied to the study of novel irrigation protocols in an in vitro model of infected root canals [17]. In this study, it was confirmed that analysis with qPCR leads to comparable results with fluorescent microscopical analysis after DAPI staining. However, a drawback of this method is that it cannot distinguish between viable and non-viable bacteria or extracellular DNA, respectively. Hence, the number of viable bacteria may be overestimated. A method to reduce the detection of extracellular DNA or DNA from non-viable organisms via qPCR is the usage of DNA-intercalating dyes such as propidium monoazide (PMA), or derivates like EMA (ethidium monoazide) or PMAxx [18,19]. PMA and its derivates are cell membrane impermeable and therefore cannot penetrate the membrane of non-compromised cells. However, in bacteria with a compromised membrane, PMA will penetrate the cell where it intercalates to DNA. In the same way, PMA will also bind to extracellular DNA. PMA can then be covalently linked to DNA by photoactivation with strong blue light [18,19]. Photolysis converts the azide group of PMA into a highly reactive nitrene radical that readily reacts with nearby organic molecules [20]. Due to the proximity of the intercalated dye to DNA, a large proportion of PMA will therefore become covalently linked to the DNA. PMA bound to DNA will then inhibit its amplification in downstream PCR reactions. A drawback of PMA-based live/dead qPCR is that the incubation conditions have to be optimized depending on targeted bacterial species and the experimental setup [21], especially the concentration of the dye, incubation time with the sample, and length of photoactivation, but other factors can also influence the quality of the assay. Excessive concentrations of PMA and elongated incubation times can result in leakage of the dye into viable cells. However, too low concentrations of PMA and short incubation times may not sufficiently stain enough DNA from non-viable cells or extracellular DNA to efficiently inhibit its amplification. A further drawback arises from turbidity or samples with low permeability for blue light, preventing sufficient illumination. 

This PMA method was successfully applied in several studies in dentistry [22,23,24]. For example, this method was used previously on *Enterococcus faecalis* (*E. faecalis*) both under in vitro conditions as well as under in vivo settings with samples obtained from infected root canals [25,26]. However, these samples were obtained by collecting specimens with paper points and by flushing of biofilms, respectively. This leads to bias by mainly harvesting easily accessible bacteria and neglecting bacteria within the ramifications and dentinal tubules. Furthermore, this does not allow for absolute quantification of bacteria within the root dentin. 

Therefore, the objective of the present study is to differentiate between viable and non-viable bacterial cells derived from the dental hard tissue by viability qPCR. Moreover, the ambition was to be able to quantify the bacteria from the complete root system containing the strongly branched side canals and dentinal tubules. For this purpose, the optimal concentration of PMA, its incubation time, and the length of blue light treatment needed to be established to reliably discriminate viable/non-viable bacteria for application on dental hard tissues for the first time. 

## 2. Materials and Methods

### 2.1. Chemicals, Teeth, and Bacterial Strains

*E. faecalis* was obtained from the German Collection of Microorganisms and Cell Cultures (DSMZ, Braunschweig, Germany). Human premolars were purchased from Enretec GmbH (Velten, Germany). In the present study, all teeth were extracted for medically justifiable reasons not connected to the research objective. No information was available on the patients’ sex, age, name, or general health condition. Ethylenediaminetetraacetic acid (EDTA), sodium chloride (NaCl), and tris(hydroxylmethyl)aminomethane (tris)-HCl were acquired from Carl Roth (Karlsruhe, Germany), lysozyme and agarose from Sigma-Aldrich (St. Louis, MO, USA), and Triton X-100 from Serva (Heidelberg, Germany). Tryptic soy broth (TSB) was purchased from Becton Dickinson (East Rutherford, NJ, USA) and PMAxx from Bioticum (Fremont, CA, USA).

### 2.2. Treatment of Purified Genomic DNA of E. faecalis with PMAxx

Purified genomic DNA of *E. faecalis* was diluted to a concentration of 20 ng/µL and PMAxx was diluted to 20 µM in ddH_2_O. Then, 20 µL of DNA was mixed with 20 µL of PMAxx (final concentration of 10 µM). The DNA/PMAxx mixture was incubated for 30 min in the dark and afterwards subtracted to blue light illumination for 30 min on a Glo-PlateBlue LED Illuminator (Bioticum, Fremont, CA, USA). As a control, an identical sample was not subtracted to blue light illumination and to another sample, 20 µL ddH_2_O without PMAxx was added.

The samples were then purified with the ReliaPrep gDNA Tissue Miniprep System (Promega, Madison, WI, USA) according to the manufacturer’s instructions.

### 2.3. Treatment of Planktonic E. faecalis with PMAxx

A culture of *E. faecalis* was grown for 16 h at 37 °C by inoculating TSB from a single colony, then subcultured into fresh TSB by diluting the culture 100-fold. The subculture was then grown to an optical density between 0.25 and 0.35 (viable fraction). The bacteria in an aliquot of this culture were killed by incubation at 100 °C for 120 min (non-viable fraction). Then, 200 µL of both fractions was then incubated with 0, 10, 25, 50, or 100 µM PMAxx for 10 min in the dark and subjected to blue light illumination for 15 min. Afterwards, genomic DNA from the samples was purified with the ReliaPrep DNA Tissue Miniprep System according to the manufacturer’s instructions. 

### 2.4. Preparation of Teeth and Cultivation with E. faecalis

Preparation of the specimens and inoculation of the teeth has been described previously [16,17,27]. The crowns of 88 human premolars were separated from the roots to ensure a uniform root length of 16 mm. Then, the roots were prepared with rotary controlled nickel–titanium instruments (ProTaper Gold F1 and F2, Dentsply, York, PA, USA) under constant irrigation with 0.9% NaCl up to a working length of 15 mm. Afterwards, the teeth were sonicated for 10 min in an ultrasonic bath in the presence of tryptic soy broth (Merck, Darmstadt, Germany) and autoclaved for 40 min at 121 °C for sterilization. Finally, the teeth were embedded in 3% agarose in 1.5 mL conical tubes.

Next, the root canals were inoculated with *E. faecalis*. A culture of *E. faecalis* was grown from a single colony for 16 h at 37 °C in TSB, then diluted to 1.5 × 10^8^ CFU/mL. The root canals were inoculated with 10–20 µL (depending on the size of the root canal) of the diluted culture of *E. faecalis* on two consecutive days and incubated for 21 days at 37 °C. The medium was exchanged daily, except on weekends.

### 2.5. Treatment of Teeth with PMAxx and Grinding of Teeth

After 21 days of colonization with *E. faecalis*, the roots were separated into 11 different groups with 8 roots within each group. The chosen control groups were 4 human premolars with viable bacteria without the application of PMAxx and 4 human premolars with non-viable bacteria without the application of PMAxx.

The bacteria within 4 roots in each group were killed by incubating the roots for 2 h at 100 °C. The medium was removed from the root canals and the root canals were filled with the indicated concentration of PMAxx diluted in phosphate-buffered saline (PBS). For a better distribution of PMAxx, the root canals were ultrasonically irrigated with IrriS (VDW, Munich, Germany) for 10 s. Afterwards, the root canals filled with PMAxx were first incubated for the indicated amount of time in the dark and then illuminated with blue light for the indicated amount of time using a Glo-Plate Blue Led Illuminator (Bioticum, Fremont, CA, USA).

Afterwards, the roots were ground by a 6775 Freezer/Mill cryogenic grinder (SPEX, Metuchen, NJ, USA) using the following parameters: precool, 10 min; run time, 1 min; cool time, 1 min; impactor rate, 12; cycles, 4. The teeth were hereby cooled with liquid nitrogen to prevent overheating. The tooth powder was then stored at −80 °C until DNA purification. 

### 2.6. Purification of Genomic DNA from Tooth Powder

As described in previous studies [16,17], 15 mg of tooth powder was dissolved in 200 µL of 0.5 M EDTA and incubated for 24 h at 37 °C under agitation. Then, the tooth powder was centrifuged at 8000× *g* for 30 s, the supernatant was transferred to a new tube (supernatant fraction), and the pellet (pellet fraction) was resuspended in 200 µL 0.5 M EDTA and incubated for another 24 h at 37 °C under agitation. The tooth powder was again centrifuged and the supernatant was combined with the first supernatant fraction. Then, the pellet fraction was resuspended with 180 µL lysozyme solution (20 mg/mL lysozyme in 20 mM tris-HCl pH 8.0, 1.2% Triton X-100, 500 mM EDTA), while 360 µL lysozyme solution was added to the supernatant fraction. Both fractions were then incubated for 72 h under agitation. Then, both fractions were mixed with 1 volume of cell lysis buffer and 0.1 volumes of proteinase K and incubated for 2 h at 56 °C. The DNA was then isolated with the ReliaPrep gDNA Tissue Miniprep System (Promega, Madison, WI, USA) according to manufacturer’s instructions. The DNA was eluted twice with 50 µL ddH_2_O [5,6].

Genomic DNA of *E. faecalis* for the generation of standard curves was also isolated from an overnight culture with the Relia Prep gDNA Tissue Miniprep System according to the manufacturer’s instructions.

### 2.7. Quantitative Real-Time PCR

A sample of 2 µL of isolated DNA from both fractions was analyzed by qPCR in a CFX96 Real-Time system (Bio-Rad, Hercules, CA, USA) with the SsoAdvanced Universal SYBR Green Supermix (Bio-Rad), with the following primers targeting the 16S rRNA of *E. faecalis*: 5′-CCGAGTGCTTGCACTCAATTGG-3′ and 5′-CTCTTATGCCATGCGGCATAAAC-3′. Then, 10-fold dilutions of genomic DNA of *E. faecalis* between 10 ng and 10 fg were used for the generation of standard curves. All qPCR reactions were performed in triplicates.

### 2.8. Statistical Analysis

To determine the differentiation between viable and non-viable microorganisms, statistical analysis of the determined values was carried out using the jamovi software version 2.3.28 (the jamovi project, Sydney, Australia) with logarithmized values. One-way ANOVA was applied to investigate the differences between different PMAxx concentrations, incubation time, and length of blue light treatment. The Games-Howell test was selected as the post-hoc test and the significance level was set at 5%. 

## 3. Results

### 3.1. Test of PMAxx for Functionality

In the first step, it was verified that treatment with PMAxx inhibits amplification of genomic DNA by qPCR. Therefore, purified genomic DNA of *E. faecalis* was treated with 200 µM PMAxx, incubated for 30 min in the dark, then photoactivated for 30 min under illumination with blue light. As controls, a second set of DNA was mixed with PMAxx but not photoactivated by blue light illumination and a third set was left untreated. The genomic DNA was then purified and dilutions of the DNA were assessed with qPCR.

**Figure 1:**
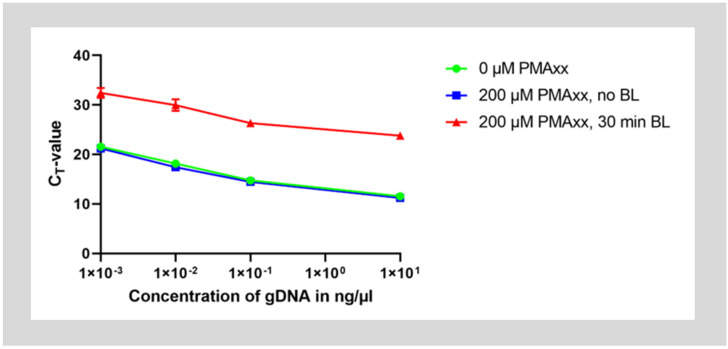
Treatment of purified genomic DNA with PMAxx. DNA was treated with 200 µM PMAxx and was either photoactivated by blue light illumination (red curve) or left without treatment under blue light (blue curve). As a control, some DNA was also left completely untreated (green curve). The DNA was then purified and qPCR was run on the samples diluted to concentrations between 10 ng/µL to 1 pg/µL.

Untreated DNA and DNA mixed with PMAxx without photoactivation showed amplification with a ΔC_T_-value of approximately 3.3 cycles between 10-fold dilutions of DNA. However, after treatment with PMAxx and photoactivation, C_T_-values for the same starting concentration of DNA increased by approximately 12 cycles. This means that about 4000 times or 2^12^ less starting DNA could be detected. Therefore, only about 0.025% of DNA was detected. Hence, approximately 99.975% of the DNA could have been inactivated by the PMA treatment (Figure 1). 

**Figure 2:**
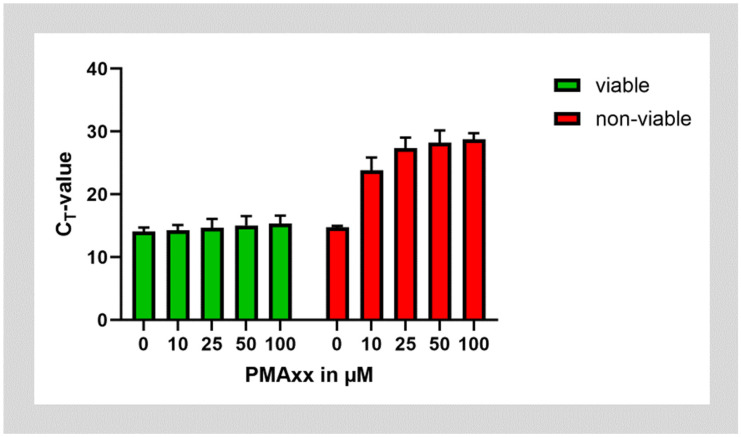
Treatment of viable and non-viable *E. faecalis* bacteria with different concentrations of PMAxx.

In the next step, we tested whether PMAxx could be successfully used on cultures of *E. faecalis*. To test PMA on non-viable bacteria, aliquots of the bacterial cultures were inactivated by incubation at 95 °C. Both batches of viable and non-viable *E. faecalis* were then treated with ascending concentrations of PMAxx. PMAxx treatment almost did not affect the amplification of *E. faecalis* DNA from viable bacteria. However, treatment of non-viable bacteria with PMAxx shifted the C_T_ value after amplification of DNA by approximately 14 cycles. This corresponds to about 99.994% of DNA being inactivated by incubation with PMAxx. Saturation was reached at a PMAxx concentration of 25 µg/mL, while higher concentrations of PMAxx did not lead to further improvement (Figure 2). 

### 3.2. Quantitative Live/Dead qPCR in Infected Root Canals

**Figure 3:**
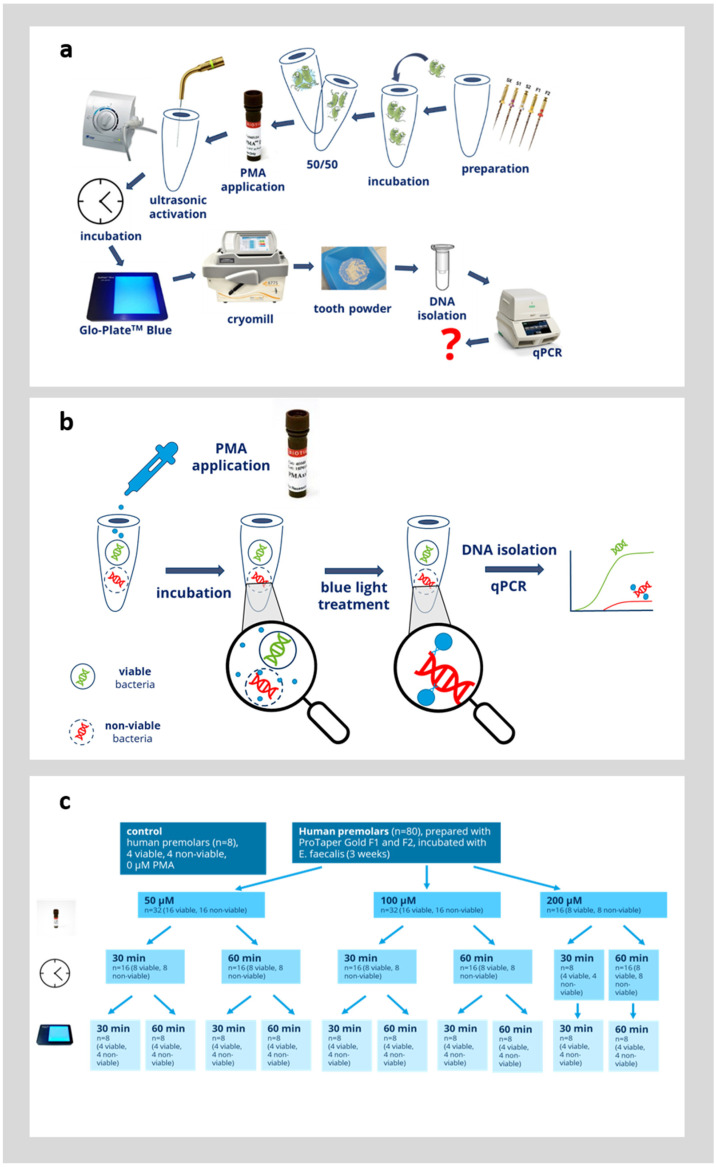
Overview of the experimental protocol. (**a**) Schematic of the complete experimental protocol. The question mark (?) in red indicates the experimental outcome of interest. (**b**) Overview of the principle of PMA treatment. (**c**) Overview of the analyzed variables of PMA treatment.

In the next step, we evaluated whether PMAxx could be used to distinguish between viable and non-viable bacteria directly in infected root canals. Therefore, an established model was used and the procedure is outlined in Figure 3a,b. Root canals were colonized with *E. faecalis* for 3 weeks, as detailed in the Materials and Methods Section. In half of the teeth in each group (*n* = 4), the bacteria were inactivated by thermal heating for 2 h at 100 °C. It was confirmed in the preliminary experiments that this is sufficient to inactivate all bacteria within the root canals [4,5]. Afterwards, the bacteria within the root canals were incubated with 50, 100, or 200 µM PMAxx (Figure 3c). The bacteria within the root canals were first preincubated with PMAxx for 30 or 60 min and afterwards, PMAx was crosslinked to bacterial DNA by blue light illumination for 30 or 60 min. As a control, teeth with viable bacteria and teeth with inactivated bacteria were left untreated. The viable control group showed the qPCR results without the effect of PMAxx and this group displayed the maximum amount of bacterial DNA detectable by qPCR. The non-viable control group showed the qPCR results without the effect of PMAxx. This devitalized control group was supposed to display the same amount of bacterial DNA detected by qPCR as in the viable group, since the PMA did not inhibit the qPCR. Afterwards, the teeth were ground by a cryomill, then genomic DNA was extracted from the tooth powder and evaluated by quantitative PCR as outlined in the Materials and Methods Section. During DNA extraction, two fractions were obtained (pellet fraction and supernatant fraction). The obtained C_T_-value was used to calculate the corresponding number of bacteria from a standard curve obtained by amplification of genomic DNA with known concentrations. The number of bacteria detected from both the pellet and supernatant fraction were added up to obtain the overall number of bacteria within each root.

**Figure 4:**
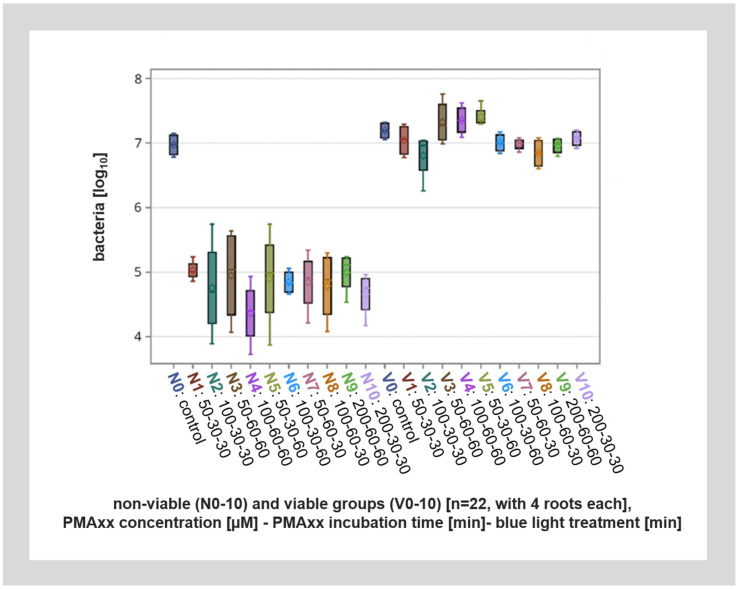
Overall visualization of the different viable (V1–V10) and non-viable (N1–N10) groups with different PMAxx concentrations, incubation times, and blue light application lengths as well as the viable and non-viable controls (V0, N0) by qPCR. The viable groups (V1–V10) as well as the viable and non-viable controls (V0, N0) show a significant difference to the non-viable groups (N1–N10).

In root canals with viable bacteria, PMAxx treatment had no significant effect on the number of bacteria detected after qPCR analysis. Also, within the control specimens without PMA treatment, similar amounts of DNA were detected from the root canals with viable bacteria and root canals with non-viable bacteria (inactivated by thermal heating). However, PMAxx treatment of non-viable bacteria lead to up to a 200-fold reduction in detected bacteria, or up to approximately eight cycles. This means that about 99.6% of DNA from non-viable bacteria was inactivated by PMAxx treatment (Figure 4).

**Figure 5:**
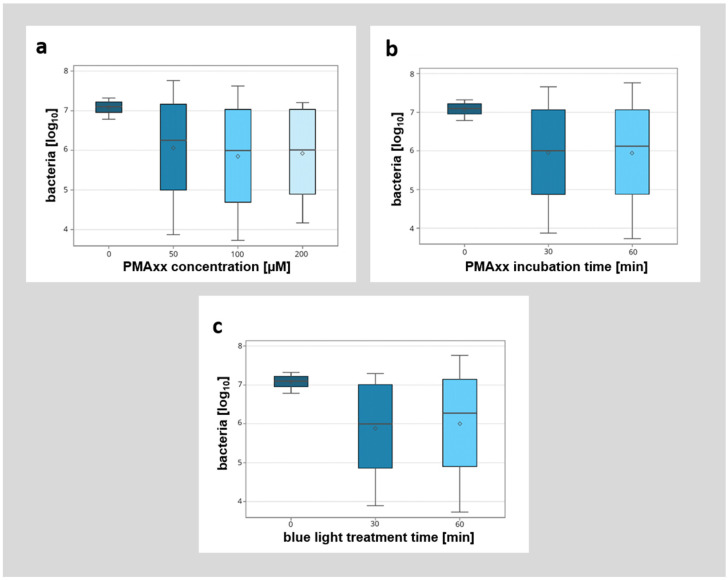
Detailed breakdown of the influence of different parameters (PMAxx concentration [µM], PMAxx incubation time [min], blue light treatment time [min]) on the detection of non-viable bacteria by qPCR. (**a**) Influence of different PMAxx concentrations (0 µM, 50 µM, 100 µM, 200 µM) on the detection of non-viable bacteria. (**b**) Influence of different PMAxx incubation times (0 min, 30 min, 60 min) on the detection of non-viable bacteria. (**c**) Influence of different blue light treatment times (0 min, 30 min, 60 min) on the detection of non-viable bacteria.

A detailed breakdown of the different modified factors (PMAxx concentration, incubation time, and duration of blue light illumination) showed no significant influence of the analyzed factors (Figure 5). However, while there was no significant difference between the different incubation protocols with PMAxx, higher concentrations of PMAxx with longer incubation times (e.g., 200 µM PMAxx concentration, 30 min incubation time, 30 min blue light treatment) seemed to allow for better discrimination between viable and non-viable bacteria. 

Overall, these experiments suggest that viability PCR with the administration of PMAxx is possible directly in colonized root canals in laboratory research. Also, a PMAxx concentration of 100 µM with a preincubation time of 60 min and blue light illumination for 60 min, or a concentration of 200 µM with a preincubation of 30 min and blue light illumination of 30 min, seemed to be optimal. These setups did not lead to a significant labeling of viable bacteria, but did lead to the lowest level of non-viable bacteria detected. Due to the shorter time required, we would thus recommend a PMAxx concentration of 200 µM with a preincubation time and blue light illumination time of 30 min in future experiments using these experimental settings.

## 4. Discussion

In a previous paper, we optimized a method for molecular biological evaluation of microbial colonization within dental root canals [16]. This method was successfully applied to the evaluation of cleansing protocols used in endodontics [17]. In the present study, this method was extended to the inclusion of a viability stain that allows for the distinction between viable and non-viable bacteria in quantitative PCR. 

Viability PCR was first established around 20 years ago using ethidium monoazide (EMA) as a DNA intercalating dye [28]. Since then, improved dyes like propidium monoazide or the proprietary PMAxx were widely used for viability qPCR. Several studies also used viability qPCR within root canals and/or for *E. faecalis* [25,26]. We could successfully adopt this method for the application within complete root canals. Higher PMA concentrations with longer incubation times and a longer duration of blue light illumination led to a more profoundly reduced detection of non-viable bacteria, although this was not statistically significant. Nevertheless, a concentration with 200 µM PMAxx and a preincubation and blue light illumination of 30 min each seemed to be optimal for the differentiation of viable and non-viable bacteria. Furthermore, a longer blue light treatment time could compensate for a lower PMAxx concentration (100 µM PMAxx concentration, 30 min incubation time, 60 min blue light treatment). It is important to note that no complete inhibition of the amplification of DNA from non-viable bacteria can be achieved. This is because PMA will not be able to inactivate DNA from all bacteria, and this observation is in line with other previous studies [25,26,29]. This generally leads to a slight overestimation of viable bacteria, especially in the presence of a large number of non-viable bacteria or free DNA. This is due to the technical limitation of PMAxx which may not penetrate all cells with an impaired membrane. Also, non-viable “ghost cells” may sometimes still have a membrane impermeable to the dye, though no longer being metabolically active [21]. Furthermore, depending on the experimental conditions, an insufficient amount of PMAxx may bind to the target DNA amplified in the subsequent qPCR reaction. Hence, while PMAxx may have penetrated non-viable cells, the fragment of DNA targeted by qPCR may not be labeled in the DNA derived from a subset of bacteria. Therefore, longer amplicons are typically better for viability qPCR. However, amplicons that are too long reduce the efficiency of the qPCR [20]. In this study, a shift of approximately 14 cycles could be achieved between viable and non-viable bacteria. This is also comparable to, or even better than, other studies [25,26]. In our study, we observed almost no inhibition of amplification of viable bacteria by qPCR even after incubation with higher concentrations of PMAxx. Other studies have often observed a stronger impact of dyes used for viability detection also on viable bacteria [26,30]. A reason for this might be a low background of non-viable bacteria, or the use of PMAxx as a stain instead of regular PMA or EMA. EMA has a higher permeability into bacteria with an intact membrane compared to PMA and therefore generally leads to a stronger reduction of detected DNA from living bacteria, especially after treatment with higher concentrations of the dye [29,31]. This is attributed to EMA having only one positive charge while PMA has two positive charges [20,31]. Information on the exact chemical composition and relationship of PMAxx to PMA is proprietary information from its manufacturer Biotium. However, according to Biotium, PMAxx offers even increased discrimination in viability PCR between living and non-living organisms [32]. This is in line with the results of this study, where a rather good distinction was achieved between viable and non-viable bacteria while almost no effect of the dye was recorded in viable organisms. 

A challenge of viability qPCR in complex samples is the accessibility of the dye to the microorganisms in complex samples, as well as the light permeability in opaque or turbid samples. A study estimated that an energy of approximately 51–82 Kcal/mol in PMA is necessary to convert the azide group to nitrene and thereby trigger the covalent binding of PMA to DNA [33]. Therefore, longer exposure times with the dye in opaque or turbid samples are typically necessary [34]. Hence, different concentrations of PMAxx, exposure times, and blue light illumination times were tested. We could show that all of the combinations tested showed similar results. As verified in the present study, the blue light definitely penetrates the hard dental tissue dentin causing the cross-linking of the PMAxx to the bacterial DNA and preventing DNA amplification. However, the influence of dentin acting as a photometric barrier could possibly lead to the reduced accessibility of the blue light to the PMAxx attached to the DNA and therefore, lead to an increased background measurement of non-viable bacteria detected by qPCR.

A further obstacle regarding the access to the bacterial DNA for the qPCR is the dental hard substance. In order to obtain to the bacterial DNA, the dental hard substance needs to be ground and a lysis protocol needs to be performed to remove the anorganic and organic parts of the substance, without destroying the bacterial DNA. In the present study, *E. faecalis* was used as a model organism for the establishment of the PMAxx protocol in dental hard substances. However, PMA has been used on several bacterial species, as well as on yeasts, fungi, viruses, and parasites [35,36]. Therefore, our established protocol can be applied to other bacterial and eucaryotic species and viruses, although the protocol may need to be slightly adapted.

The establishment of this method will be a very valuable tool for the evaluation of root canal irrigation protocols, medical dressings, and even preparation systems in endodontic laboratory research. With this method, a sensitive and rapid viability analysis can be performed. The steps of time-consuming culturing, microscopy, or colony counting will not be not necessary. Furthermore, it will allow the detection of viable, non-culturable (VNBC) species that cannot be cultured with current technology [37]. In addition, a very important novelty of the established method is the ability to quantify the bacteria of the complete root canal, since the complete root is ground and the bacteria are distributed evenly. With the knowledge of the root weight and the amount of bacterial DNA in our analyzed sample, we were able to calculate the amount of viable and non-viable bacterial DNA in the complete root instead of only analyzing parts of the root, and thereby missing a subset of present bacteria in the dentinal tubules or the branched root canal system. However, complementary methods like microscopical analyzes or colony counting delivers other pieces of information. So, depending on the research question, viability PCR and other methods for the analyses of infected root canals should be used to compliment this method. This protocol is difficult to implement in clinical settings as the complete root needs to be ground for analysis. So, this method is mostly useful for basic endodontic and laboratory research. However, an aspect to consider in clinical settings, where our established method could be beneficial, is the application on teeth extracted due to justified medical reasons, e.g., the application for microbiome analysis by next-generation sequencing applications in different pathologies (e.g., irreversible pulpitis or apical periodontitis). 

## 5. Conclusions

In summary, we successfully adapted protocols for viability qPCR for the application of this method directly within dental hard substances—especially in the root dentin and within the root canals. This method can be used for the evaluation of different endodontic cleansing protocols, as well as for the evaluation of novel and existing root canal preparation instruments in endodontic experimental research. 

Also, the evaluation of carious dentine gained from coronal cavities can be performed easily, offering new insights into treatment strategies in operative dentistry. Furthermore, it can also be easily adapted for the study of microbial communities within extracted teeth and other biological hard substances (e.g., bone, cartilage, or substitutes for bone).

## Data Availability

The raw data supporting the conclusions of this article will be made available by the authors on request.
